# The lived experiences of tuberculosis survivors during the COVID-19 pandemic and government lockdown in South Africa: a qualitative analysis

**DOI:** 10.1186/s12889-023-16657-w

**Published:** 2023-09-05

**Authors:** Ann Scheunemann, Aneesa Moolla, Constance Mongwenyana, Neliswe Mkize, Mohammed Rassool, Vuyokazi Jezile, Denise Evans

**Affiliations:** 1 Health Economics and Epidemiology Research Office, Faculty of Health Sciences, University of the Witwatersrand, Johannesburg, South Africa; 2 Department of Psychiatry, Boston Medical Center, Boston, MA USA; 3 TH Chan School of Public Health, Harvard University, Boston, MA USA; 4 School of Public Health, University of the Witwatersrand, Johannesburg, South Africa; 5 Clinical HIV Research Unit, Helen Joseph Hospital, Johannesburg, South Africa

**Keywords:** Tuberculosis, COVID-19, Coping, South Africa

## Abstract

**Background:**

Tuberculosis (TB) is a major health concern in South Africa, where prior to COVID-19 it was associated with more deaths than any other infectious disease. The COVID-19 pandemic disrupted gains made in the global response to TB, having a serious impact on the most vulnerable. COVID-19 and TB are both severe respiratory infections, where infection with one places individuals at increased risk for negative health outcomes for the other. Even after completing TB treatment, TB survivors remain economically vulnerable and continue to be negatively affected by TB.

**Methods:**

This cross-sectional qualitative study, which was part of a larger longitudinal study in South Africa, explored how TB survivors’ experienced the COVID-19 pandemic and government restrictions. Participants were identified through purposive sampling and were recruited and interviewed at a large public hospital in Gauteng. Data were analyzed thematically, using a constructivist research paradigm and both inductive and deductive codebook development.

**Results:**

Participants (*n* = 11) were adults (24–74 years of age; more than half male or foreign nationals) who had successfully completed treatment for pulmonary TB in the past two years. Participants were generally found to be physically, socioeconomically, and emotionally vulnerable, with the COVID-19 pandemic exacerbating or causing a recurrence of many of the same stressors they had faced with TB. Coping strategies during COVID similarly mirrored those used during TB diagnosis and treatment, including social support, financial resources, distraction, spirituality, and inner strength.

**Conclusions:**

Implications and suggestions for future directions include fostering and maintaining a strong network of social support for TB survivors.

## Background

Tuberculosis (TB) is one of the top public health concerns in South Africa and is responsible for high rates of deaths [1, 2]. In 2020, an estimated 328,000 people were confirmed ill and 61,000 died because of TB [3, 4], making South Africa among the top five countries that struggle with TB globally. Comorbidity with the Human Immunodeficiency Virus (HIV) epidemic contributes to the country’s TB crisis [5] and of the 61,000 estimated deaths related to TB in 2021, 36,000 deceased were estimated to be HIV positive. Sociocultural factors including economic insecurity and stigma [6] compound the effects of physical diseases and contribute to the high prevalence [7, 8] and disruption of care [9]. Most South Africans live in areas that are subjected to high levels of poverty, unemployment, poor housing, and sanitation [10]. Mental illness and substance use place additional burdens on TB patients, with one recent study finding an 82% prevalence rate for mental disorders, including depression, anxiety, and substance use, among participants [11]. All these factors have a role in driving the status quo of TB in South Africa.

Remedial strategies have been implemented to mitigate the spread of TB and offer socioemotional support to patients and survivors. However, the emergence of the COVID-19 pandemic has stunted the progress of controlling TB and its effects, not only in South Africa but globally [12–17]. COVID-19 shares similar symptoms with TB, which complicated the public health response to both diseases [18] and resulted in higher fatality rates in co-infected patients [19]. Both are also influenced by the same social determinants [20] which could compound stresses in an already-vulnerable population [21]. To date, 4,055,656 positive cases of COVID-19 and 102,595 related deaths have been reported in South Africa [22]. The severity of the COVID-19 pandemic resulted in it being prioritized over TB, which led to healthcare workforces, finances, and infrastructure being redirected to mitigate the COVID-19 global burden [14, 23, 24] and resulted in deleterious impacts to patient access to healthcare and research, and consequent TB control in South Africa [25, 26].

As part of its strategy for combating the COVID-19 pandemic, the South African government declared one of the strictest lockdown measures in the world beginning in March 2020 [27–30]. The implementation of the lockdown in South Africa subjected people living with TB to various adverse effects, which impeded their access to health care services, treatment, employment, food, and economic, and psychosocial support [31–33]. The COVID-19 pandemic also contributed to reduced rates of TB diagnosis and delayed proper therapeutic decision-making due to confusion around the diagnosis of the two diseases, lack of transport for people to visit healthcare services, people’s fear of contracting COVID-19 at the healthcare centers [34], and fear of related stigma [35]. Treatment initiation was reduced [26]. Patients also experienced increased rates of negative affect including depression, hopelessness, anxiety, sadness, fear, and loneliness during the COVID-19 lockdown [34, 36, 37].

Though there is ample literature on stress and coping during COVID, few studies have examined the effect of the interaction of the COVID-19 pandemic and South Africa’s particularly severe lockdown on TB survivors, from their perspectives. Because of their increased physical and social vulnerability, it is particularly important to understand the resources and limitations of this population in this context, so that information can be applied to better support survivors in the future. The current study examined the perceptions and experiences of TB survivors during the COVID-19 pandemic and lockdown using a subset of patients from a larger, ongoing study [38, 39]. Specifically, we explored participants’ experiences of TB diagnosis and treatment, the impact of the pandemic and lockdown on their mental and physical care, and coping resources that helped them manage their stressful experiences.

## Methods

This was a cross-sectional qualitative study that formed part of a larger longitudinal study in South Africa. TB Sequel (NCT03251196) is a multi-country, multi-center, observational cohort study designed to understand the pathogenesis and risk factors of long-term sequelae of pulmonary TB in South Africa, Mozambique, Tanzania, and The Gambia [39]. Patients (≥ 18 years) with drug-sensitive pulmonary TB were recruited at TB treatment initiation between 09/2017 and 02/2020. Eligibility criteria also included willingness to provide written informed consent, willingness to be tested for HIV, consent to collection of biological materials, willingness to begin TB treatment, and living in the area. Any patients who had started anti-TB treatment in the past 6 months, had a severe medical or psychiatric condition, were incarcerated, or were participating in another study related to lung disease or TB, were excluded. Patients enrolled in TB Sequel were treated according to the local standard of care by the respective National TB program and were followed for up to 24 months.

Participants were identified through purposive sampling. Participants included TB survivors enrolled in the larger TB study at a major public hospital in Johannesburg [39], who had successfully completed TB treatment in the past two years and who had experienced the COVID-19 pandemic in South Africa, as COVID-19 survivors, as caregivers for loved ones with COVID-19, or as residents experiencing the lockdown imposed by the South African government [40–42]. Participants with moderate-to-severe baseline scores on the Kessler Psychological Distress Scale (K10) [43, 44] were prioritized, as were participants with diagnosed cases of COVID. The K10 includes 10 items related to psychological distress which are scored between 0 and 5. Sum scores between 25 and 29 suggest moderate psychological distress, while sum scores of 30 and above represent severe psychological distress.

Once a sample pool was established, participants were contacted by telephone and invited to participate. In-person interviews at the public hospital were then scheduled with interested participants and were conducted in a private room in the preferred language of the participant – English, isiZulu, or Sesotho. One participant was unable to meet in person and so a telephone interview was scheduled instead. Participants were asked about their experiences of stress during the COVID-19 pandemic, coping strategies to alleviate stress and promote well-being, experiences of physical and mental healthcare provision, and recommendations for improving healthcare. Interviews were conducted between November 2021 and July 2022 and lasted from 30 to 75 min. The study was approved by the Human Research Ethics Committee at the University of the Witwatersrand, Johannesburg (Clearance number: M160971).

All interviews were recorded, with participant informed consent. Facilitator field notes were also used to summarize the interviews and record details such as participant mood and mannerisms and environmental characteristics. Researchers translated and transcribed the interviews into written English, after which the transcriptions were coded and common themes extracted, using a constructivist research paradigm with content analysis [45] in NVivo 11. Coding was completed using a codebook that was developed a priori, and to which additional codes were added inductively after an initial read of the data. Two researchers coded and compared findings for intercoder reliability. Coded data were then extracted, and themes were developed.

Major themes common to multiple participants were extracted and presented through the aggregation of findings into two composite vignettes that reflect the experiences of TB survivors. Vignettes as a method for presenting results have gained popularity in recent years [46, 47], and composite vignettes have been used to exemplify a representative scenario of the research topic [48] and have been found by research participants to be trustworthy to their experiences [49] while also maintaining the anonymity of the participants [46]. The names used in the vignettes below do not reflect the names of any participants in the study.

## Results

**Fig. 1:**
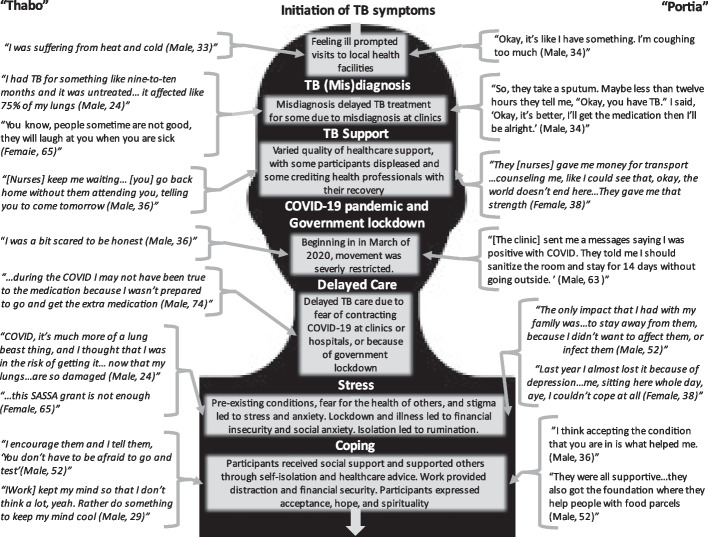
Pathways of experiences, stress, and coping in TB survivors

An initial participant pool of 62 was developed. Ten TB survivors participated in in-person interviews and one TB survivor participated in a telephone interview. Recent research has confirmed that eleven participants are considered adequate for reaching saturation [50]. Reasons for non-participation included that the potential participant was deceased, unable to or not interested in participating, or inaccessible. Five participants were South African citizens; the other six participants were residents of South Africa who had immigrated from other sub-Saharan countries. Eight participants identified as male. Ages ranged from 24 to 74, and occupations included part-time and full-time employment as a computer programmer, hair stylist, mechanic, construction worker, or upholsterer. Most participants experienced some disruption in employment due to the COVID-19 pandemic and government lockdown, meaning that they were unemployed for at least part of the pandemic. Pathways representing experiences of stress and coping in TB survivors during COVID can be found in Fig. 1. After the vignettes, common themes described by study participants are further outlined.

### Portia

Portia is originally from another sub-Saharan African country and moved to Johannesburg approximately twenty years ago for work. She is HIV-positive and in 2017 began feeling sick, became increasingly weak, and lost weight. She was quickly diagnosed with TB and began treatment. Because of her symptoms, she was unable to work but had friends nearby who supported her financially by providing groceries, physically by collecting her medication for her, and emotionally by phoning her to check in and visiting to chat through the window. Portia was also grateful to the healthcare workers, whom she experienced as patient, kind, and informative, and who ensured that she was able to attend medical appointments and access medication. She feared stigma related to TB and so limited the number of people who knew about her condition to those she felt she could trust.

Once her health improved Portia was able to find employment again, but her work became unsteady once more with the advent of the COVID-19 pandemic. Her past TB continued to limit her physical functioning, sometimes accompanied by shortness of breath and difficulty breathing, weakness, and fatigue, which caused a decline in her health and placed her at increased risk for recurrent TB. Additionally, because she was not a South African citizen she was unable to receive government financial assistance and so tried to adapt by selling her household items on the street, a task further complicated by police officers who were enforcing the government’s strict lockdown by chasing people off the streets. Portia was able to continue attending doctor’s visits and collecting her medication because some of the staff at the primary health clinic, with whom she had become friendly, offered her financial assistance. As she was living alone, the lockdown left Portia isolated, which came with mixed feelings. On the one hand, she was afraid for herself because of her HIV status and heightened risk as a TB survivor, so she felt the isolation could protect her from contracting COVID. But the lockdown also brought loneliness and the closed borders separated her from her family. Financial insecurity further isolated her from her family, as she struggled to pay for the data to be able to even phone home. She tried to keep herself busy at home by finding distractions through reading, exercising, and hobbies, which minimized her frustration.

Despite her caution, Portia contracted COVID and further isolated herself so that she could protect others. She had also heard of people with COVID being stigmatized and wished to protect herself from the judgment of others, and so quarantined alone. She began to talk to herself, leading to overthinking and worry and became anxious around people. Despite her fears, she was deeply grateful for her supportive church group and neighbors, who would bring her food and medicine, and check on her by talking through the windows of her flat. The long quarantine and illness left Portia feeling anxious and depressed, which she attempted to alleviate through prayer and hope that the pandemic would one day pass….

### Thabo

Thabo was born and raised in Johannesburg and has a wife and child. In 2019 he developed a persistent cough and felt feverish. He visited his clinic multiple times and was repeatedly misdiagnosed. His symptoms increased, and he began losing weight. Months later he switched health facilities and was finally correctly diagnosed with TB, but by that time most of his lungs had been affected. He was disappointed with the healthcare he received even after his diagnosis and felt that the doctors and nurses were not communicating with him adequately about his condition and treatment, and how to protect his family. He compared this with his HIV care. Having tested positive for HIV years prior, he had regular clinic check-ups in which healthcare workers would tell him about his condition, but at which he also met many other people living with HIV and was able to receive support and advice from them. Because the COVID-19 pandemic and lockdown began soon after his TB diagnosis, he was unable to interact with TB survivors in the same way. The government lockdown made it difficult to attend his medical appointments. Even when he did come for check-ups, social distancing prevented him from conversing with other patients as he had before. Additionally, he was afraid that going to the primary health clinic would increase his susceptibility to COVID-infection, because of the number of patients also attending appointments and the transmissibility of the disease. His fear caused him to delay getting prescriptions refilled and going to clinical check-ups. Despite this, his condition continued to improve slowly, though he still struggled with weakness and shortness of breath and suffered side effects from his medication. He was frustrated at his inability to be as active as he once had been. Lack of money was another reason for non-adherence, as he was not working and therefore was unable to pay for transportation to his primary health clinic. His family struggled to afford food and he was stressed that even his young child recognized the difference in their lifestyle. Despite increasingly limited formal work, Thabo was eventually able to pick up informal work intermittently, which helped to subsidize the government financial support that he felt was inadequate. The work also provided the additional benefit of being a distraction during the pandemic. He would work in his backyard when concern for his family’s well-being became so great that he feared arguing with his wife. He was also able to remain in contact with his parents and siblings, though he struggled at times to pay for mobile data. His family and friendly neighbors provided each other with social support. At times they would congregate informally in a small common yard while maintaining social distancing. They also helped one another with groceries, as they all struggled with finances at different points during the lockdown. Thabo would even occasionally collect medication for neighbors who were unable to attend the clinic to fill their prescriptions. Before the COVID pandemic and lockdown, Thabo had found it personally rewarding to advise friends, colleagues, and other people he met who displayed symptoms he recognized as potentially related to TB or HIV, by telling them that they did not need to be afraid and by encouraging them to get tested. Because he had appreciated those patients who had advised him on living with HIV when he was diagnosed, he wanted to extend that to other people he met in his life. He also had never felt stigmatized when disclosing his HIV and TB status, which gave him the confidence to be open and unafraid when discussing his illnesses with others. The government lockdown hindered his ability to provide this support, and so limited this important coping strategy, though as the lockdown has eased over time he has once again been able to provide support to others.

### The patient journey

Portia and Thabo’s stories reflect the different pathways to healthcare that this study’s participants experienced on their journeys from illness to diagnosis to treatment. Additionally, their individual tales portray common experiences during the COVID-19 pandemic, as participants navigated this infectious respiratory disease and consequent government lockdown. Exploration of participant experiences is detailed chronologically below, from the initiation of TB symptoms, diagnosis, and treatment through to the COVID-19 pandemic as a TB survivor. In particular, participant stress and coping strategies during these periods are highlighted.

#### Initiation of TB symptoms and TB diagnosis

"Somedays I will wake up with sore legs and I can’t walk. Somedays my hands I was feel like I was lifting a heavy stuff, like I have pains like in my toes, in my waistline…."

Upon initiation of symptoms, both Portia and Thabo sought help at their local health facilities. Despite displaying symptoms common to TB, and despite the prevalence of TB in South Africa, Portia and Thabo had vastly different experiences in accessing healthcare for their illness. Portia was quickly diagnosed and immediately begin treatment, similar to the experiences of some study participants: “At the clinic, they took sputum. When I was thinking of going back to collect my results, then COVID attacked me and then the results came positive for TB, [healthcare workers phoned, saying] ‘We will come to collect you,’ then they come,” (Female, 65).

Thabo was initially misdiagnosed and therefore his care was delayed for months. Multiple participants had a similar experience: “They took sputum. They told me to come after three days, after three days they told me they can’t find results. They take it again. For almost three weeks,” (Male, 36). Thabo’s misdiagnosis also meant that for months he was interacting with, and possibly transmitting and infecting other people. As his condition did not improve, he eventually sought healthcare elsewhere and was finally correctly diagnosed, by which time his condition had significantly worsened. Even then, Thabo was dissatisfied with the care and communication from the doctors and nurses and concerned for the wellbeing of his family. As expressed by one participant: “You come here, you get your checks, they do the sputum, they do everything that is needed, send you to x-rays, and you don’t get any feedback,” (Male, 74). Participants in fact described differing qualities of healthcare, with about a third of participants expressing that their healthcare workers did not provide them with adequate information regarding their diagnosis or progress, counsel them sufficiently on how to prevent transmission to loved ones, or follow-up to check their condition and offer support. This lack of communication was frustrating, a feeling compounded by the infrastructure of the health system; in Thabo’s case, he would at times attend the clinic and wait all day without seeing a medical practitioner and then be asked to return the next day.

This contrasts with Portia’s experience, who felt that the healthcare providers were very sensitive, supportive, and informative throughout her treatment, as exemplified by one participant: “After 21 days I had power and realized that doctors know their job,” (Male, 63). In fact, even participants who were displeased with healthcare services expressed positive feelings towards particular nurses and doctors who were supportive: “Well, you know in the beginning, uh, there was a really nice sister. She was, uh, really very, very awesome. Uh, she cared. You could see,” (Male, 74). In this way, participants differentiated between an individual healthcare provider and the healthcare system itself.

For both Portia and Thabo, their diagnosis was life-changing. They experienced long-term physical impacts, similar to other participants: “Because of the limitations that I have in doing most activities, that health. I sometimes have, like, your mental breakdowns, your emotional breakdowns,” (Male, 24). The stress of unemployment and consequent economic strain was also common: “When I had TB I really, it was hard because I couldn’t go to work,” (Male, 29). A TB diagnosis also resulted in social consequences. For Thabo, he was concerned for the well-being of the loved ones with whom he was living and felt that the healthcare providers were more concerned with data collection and research than helping him devise ways to protect his wife and child. Despite this stress, participants found some support from family members who were helping to care for them during their illness: “Yeah, like it would, better, like it would better things. It would. Because of, I would not feel alone, I wasn’t feeling alone with them around. Like I felt like, okay, like I had someone to hold, hold my hand throughout this whole healing journey or recovering journey, so. It felt great,” (Male, 24).

Portia found strength in the medical, material, and emotional support offered by the primary health care nurses who were caring for her, who gave her medication, counseling, and financial support for transportation. Outside of the health facility, though, she was wary of judgment and so practiced self-care in choosing only to disclose her status to trusted loved ones. In this way, she managed to avoid judgment but also further isolated herself socially, as exemplified by one participant: “Because of, uh, if I had, like, just to, to remain around, like people and be indulging their stigmas, that would break me. So, I had to isolate myself. I had to keep a positive mind. I had to take my treatment and take good care of myself. That helped me to get through TB,” (Male, 24).

#### COVID-19 pandemic and delayed care

"Life became so hard. Very hard. If I was someone who is like, weak, I could have killed myself."

As their health improved, Portia and Thabo were able to return to work. Subsequently, with the onset of the COVID-19 pandemic, the South African government declared a strict lockdown which restricted their movement and resulted in both once again becoming unemployed. Thabo was able to receive government financial assistance and found some informal work, but, because she was not a citizen, Portia could not receive government assistance. She did try to sell her household items on the street but was threatened with arrest by the police, for breaching the lockdown. Many participants received food parcels and financial support from other sources, including family and friends, local organizations, churches, and businesses: “And I think our church also registered us by Checkers (a national grocery store) because there was a 400 Rand voucher that I got on my phone,” (Male, 52). Social workers also played a role in providing material support: “But we were lucky because social workers were giving away food parcels and it made a difference,” (Female, 65).

The pandemic also strained healthcare resources, causing further delays in scheduling appointments and preventing access to medication. At times participants struggled to access transportation to collect medication or were ill and asked friends, family, or neighbors to collect their medications. Even when transportation was available, healthcare facilities were overwhelmed with the pandemic and so wait times to receive care were extended. Additionally, some participants were afraid to go to healthcare facilities, where they felt their risk of contracting COVID-19 was higher.

#### Stress and anxiety

"Almost everyday wake up in the morning, try to think what if the person I’m living with passes or what if I pass because my body is not that much strong too, because [of] other sickness?"

Social isolation recurred for Portia and Thabo during the pandemic, as the government lockdown restricted movement. This was particularly painful for Portia, who was living alone and unable to visit her family because of the closed borders. She did interact minimally with neighbors, but also when she was diagnosed with COVID she chose to self-isolate out of fear of stigma, which resulted in poor mental health, with symptoms including depression and rumination. As one participant stated: “Because when you have, like COVID, you isolate yourself, no one can see you, that’s a time you feel very lonely. Very, very, very lonely. It seems like you can just go out and scream, ‘Yes, I’m positive. Someone come and visit me please,’” (Male, 52).

Unemployment or underemployment also caused stress, through financial insecurity. One participant explained that his status as a TB survivor was stigmatized, making him more vulnerable at work: “I was in fear of losing my employment. Because of my employers were aware that, um, I had TB, and that I am vulnerable to getting COVID. So, they made us sign some sort of form or document that, okay, please sign this as an acknowledgment form that, um, you know, that your health issues and that you might contract COVID. Because of, at the end of the day I had to ensure that, um I provide for my family. I have to buy groceries, buy clothes and all that,” (Male, 24). Most participants lost employment, at least temporarily, during their recovery from TB, COVID-19, or both, and were therefore isolated at home and tended to ruminate, which increased stress and worsened mental health: “Sickness, being sick whilst you are home doing nothing and having no support was really stressing me,” (Male, 63).

Portia and Thabo were afraid that, because of their pre-existing conditions of TB and HIV, they were more susceptible to COVID-19. Both were aware that, like TB, COVID-19 affected the lungs. Thabo did not want to go to the hospital and at times would miss appointments, resulting in delayed care and lapses in medication. This was a common fear amongst participants, which prompted many of them to restrict their movements during the pandemic, including limiting health-seeking behaviors: “What must we do? Go to the hospital?” This is a place I avoided [laughs] because it was like, you come to…whatever hospital, the virus like, live in hospitals…” (Male, 52).

Participants also feared for the well-being of their loved ones. When sick, they tried to protect others from illness: “We need to do is to stay away from other people just to protect them because being outside with COVID, you’re infecting others,” (Male, 34). They also tried to respect the emotional boundaries of their loved ones, recognizing that the pandemic was challenging for everyone: “Especially with COVID, we all had problems. No one was, was out of the, the water. Everyone, every one of us was in the water, drowning. So, uh, I was thinking to myself, ‘Let me try and make a plan for myself instead of bothering whoever I need to bother,‘” (Male, 52). This fear was compounded when ruminating on the effect their death might have on loved ones: “I’ve still got a child to raise, because she’s still now at school. I don’t wanna die now,” (Male, 52).

#### Coping

"You must take out the pain by laughing."

Despite the stress, both Portia and Thabo were able to find material support (including financial support and groceries as discussed above) and emotional support from family and friends. Non-judgmental relationships made participants feel less isolated, afraid, depressed, and anxious: “The way that they were talking to me, the way they were treating me, it was giving me that thing, okay, this is my people, this is my family,” (Male, 34). These social connections established trust, allowed the participants to feel loved and valued and helped them to believe that they could survive illness and the pandemic.

Portia also received support from healthcare workers, who provided non-judgmental encouragement and counseling: “Like, they were treating me like a brother to a sister or like brother to brother. Just like that…it makes me feel like, okay this is nothing. I’ll be healed and I’ll be sharp. I’ll be just like anyone else outside who’s not sick, who’s not having HIV, who’s not having TB,” (Male, 33). Multiple participants noted that healthcare workers would call them to check in and encourage participants to adhere to medication and appointment schedules. Healthcare workers also provided information on participants’ diagnoses, treatment, and lifestyle changes to help them manage their illness: “No, like, when you get, when I get information like, ‘Go eat like this,’ someone telling me, ‘Go do this, go and exercise,’ if I take that information into my mind, then practice it, then, I think, everything goes well. You gain,” (Female, 38).

Participants noted that distractions were useful in alleviating stress, by keeping them busy. Work, in particular, was useful, as one participant explained: “I can work ‘til I don’t know what time is it. ‘Cause, like, I’m worried, so just to keep myself busy, just work, work, work, work until I don’t know,” (Male, 29). Though the pandemic increased unemployment which complicated participants’ ability to work formally, participants sought out informal or part-time employment or engaged in hobbies, to keep themselves occupied. One participant began welding in his backyard, which provided a creative outlet that alleviated his frustration, which he also recognized improved his relationship with his wife and child. Another participant established a hair-styling business in her home. These ventures, therefore, offered both emotional and financial support. Some participants also used exercise, reading, listening to music or podcasts, and going on long drives as distractions, “Just to get my head again,” (Male, 36).

Both Thabo and Portia were able to accept the reality of the COVID-19 pandemic and find optimism about the future as the pandemic subsided. In fact, positivity helped participants to both recover from illness and survive the pandemic and lockdown: “In my mind, I had hope that there are chances that I will also survive,” (Male, 63). Spirituality also provided inner strength and physical support for Portia, who found comfort in prayer and received groceries from her church group. As one participant noted: “It’s not everybody’s belief, but to the man upstairs I’m grateful. And I’ve got a lot of gratitude every morning when I wake up – I’m still alive,” (Male, 74).

Only a small minority of participants reported negative coping strategies associated with their TB or COVID-19 experiences. One participant commented that his alcohol consumption increased when he was socializing during the pandemic.

## Discussion

In this qualitative study, which was part of a larger longitudinal study we not only explored the patient journey from the onset of symptoms until the completion of TB treatment but also the experience of TB survivors during the COVID-19 pandemic. Participants reported that symptoms commonly associated with TB prompted visits to local health facilities. Quality of care and speed of diagnosis varied, with some participants reporting multiple misdiagnoses at multiple providers before finally being diagnosed, which delayed treatment. All participants eventually attended the same clinic, where they consented to participation in the longitudinal study of which the current study was part.

TB results in physical and socioemotional impacts. Physically, both during treatment and after participants felt weak and short of breath, and side effects of medication at times added to the physical effects of the disease. Also, as noted by Loveday et al. [37], TB diagnosis and treatment “were major life disruptions” with “multiple long-term consequences,” beyond the physical. Participants additionally reported at times facing socioeconomic stressors including unemployment and financial insecurity, social isolation, and stigma. In fact, TB is known to generally affect the most vulnerable of society [6]. Socioeconomic status (SES) is considered an important indicator of the burden of HIV, TB, and COVID-19, meaning that individuals of lower SES (like many of our participants) are more vulnerable to contracting these diseases and more impacted if they do [21]. Additionally, the stigma associated with TB can increase challenges in finding employment, creating a dynamic in which health status impacts employment and employment impacts health status in an exacerbating loop that delays recovery. Participants reported at times struggling to pay for transportation, which limited access to healthcare, or to pay for food required to pair with their medication. This feedback loop can be compounded by worsened mental health. Unemployment, underemployment, and poverty in TB patients has been associated with multiple mental illnesses, including the anxiety, social anxiety, and depression noted by participants in this study [11]. Many resources devoted to diagnosing, treating, and supporting people with TB were repurposed during COVID-19, removing supports that had alleviated social and financial strain and renewing vulnerabilities. In preparing for future pandemics, officials and experts should consider the impact of resource re-allocation on the most vulnerable and develop policies to address co-occurring health crises and their social determinants, particularly as the resource reallocation during the pandemic resulted in an increased risk of co-occurring diseases that left the most vulnerable more at risk of contracting COVID-19.

Financial relief to break this cycle was mainly derived through social support. While Vanleuuw et al. [31] found that family support, in particular, may protect survivors from hunger, this study found that, in addition to family, neighbors, church groups, and at times even primary health clinic nurses provided financial assistance or food to participants, demonstrating an expanded network of social support within our sample. It is possible that because many of the participants in this study were migrants and therefore living far from family, accessing financial support was more difficult. Additionally, participants who were migrants had come to South Africa mainly in search of work opportunities, and therefore relatives did not have additional financial support to provide. Also, as noted by Vanleuuw et al. [31], the COVID-19 pandemic was financially devastating for many households, further limiting the support that family members could provide. Participants in this study may therefore have sought assistance from a broader social network, as their primary source of support collapsed. Participants also leaned on friends, family members, and neighbors for support, including collecting medication and emotional support. Families provide financial support to TB patients, but they are also often a primary source of emotional connection, providing positivity and encouraging adherence to medication [51].

Social support is a well-recognized coping strategy and can provide emotional, cognitive, and spiritual buffers from stress, in addition to being a financial buffer. TB patients found relief from interactions with other patients and with nurses [37]. Within the current study, nurses provided some participants with medical support and information about TB, and participants also noted receiving financial assistance and emotional support which fostered inner strength, thereby strengthening their resilience network [52]. Supportive health professionals were greatly appreciated and provided security and instilled trust in the health system; participants who felt unsupported expressed frustration and a lack of trust in the efficacy of their treatment. Loveday et al. [37] and Daftary et al. [51] similarly noted that participants in their study valued support from other patients, which eased isolation and through which they received emotional support and advice during their recovery. Additionally, Daftary et al. [51] note that some participants viewed connection to other patients as a silver lining during hospitalization, where they received more sympathy than they were afforded at home. Loveday et al. [37] noted the development of WhatsApp support groups which could in the future be integrated into mHealth technologies. Unfortunately, because of the social distancing imposed by the COVID-19 pandemic, participants in this study were unable to interact with other patients. One participant in this study did, however, compare his experiences of social distancing as a TB patient with care received years prior when he was diagnosed with HIV. He had appreciated the advice he received from other HIV-positive patients on coping with the disease and regretted having no similar access during his TB treatment. Future research should examine any impacts that the loss of this coping strategy may have on patient wellbeing and the potential of technology such as messaging apps to alleviate the isolation felt by TB patients. Future public health policy could consider strengthening socioemotional support systems between patients. Additionally, future research and policy should focus on improving supportive communication between medical providers and patients, to ease the mental stress of illness that can in turn worsen health outcomes.

The COVID-19 pandemic brought additional stress to the participants, as it followed a similar trajectory in exploiting physical, financial, and socioemotional vulnerabilities as TB. Many participants feared their susceptibility due to their pre-existing conditions. In fact, TB-COVID comorbidities have been found to increase health risks, and this is further exacerbated in many participants by their positive HIV status [6]. Some participants consequently expressed fear in going to health facilities, where they felt they would be at higher risk, and therefore delayed care. Many people reported avoiding hospitals and clinics during the COVID-19 pandemic. As noted above, these comorbidities intersect with mental health and socioeconomic outcomes, complicating recovery.

In addition to health concerns, many participants lost employment at least temporarily during COVID. The severity of South Africa’s lockdown and the consequent economic impacts are well documented [40, 41, 53] and resulted in renewed financial and food insecurity [31, 53]. To counter the effect of unemployment, participants leaned once again on social support systems, including religious groups, family, and neighbors to provide groceries and other supplies. Some participants found informal work, though that could risk arrest, as police were stopping people who were on the streets during lockdown without government authorization [42].

Lockdown also prevented participants from accessing previous supports and coping mechanisms. COVID-19 lockdown policies damaged social support systems [54], and while some participants had isolated themselves from loved ones during the beginning of their TB treatment the duration was shorter. This is especially concerning given the additional psychological impacts attributed to the COVID-19 pandemic on TB patients, who tend to already be vulnerable [55, 56]. The lockdown further resulted in isolation and consequent rumination. TB alone can be an emotionally and physically isolating experience, and the specter of COVID-19 reignited fears of stigmatization and social anxiety [55, 56].

Paradoxically, some participants chose to self-isolate during the COVID-19 pandemic, citing fear of their increased risk, their desire to avoid stigma, and their wish to protect loved ones, findings which support results of a previous studies of TB patients [51] and TB patients during the COVID-19 pandemic [6]. COVID-positive participants in particular noted a tension between wanting to isolate to protect loved ones and to follow the rules, but also feeling lonely and desiring company. And even when the isolation was self-imposed, participants struggled with overthinking and negative affect. This suggests that, though isolation is often considered a stress and though social support is protective, this relationship is nuanced warrants further exploration. Future studies should examine the contexts and mindsets in which isolation may be beneficial.

Participants who were able to find informal or temporary work found distraction to be a soothing counter to rumination and family strain during the COVID-19 lockdown. Distraction is a common coping strategy used by TB patients [57, 58] and among people living with chronic conditions during the COVID-19 pandemic [59, 60], though its effectiveness in alleviating stress is unclear. Participants in this study found distraction to be useful, at least in the short-term, for preventing rumination and family arguments, or as enjoyable activities. It is possible that any benefits of distraction lie in its role as a moderator, to either decrease harmful behavior (e.g., rumination, arguments) that lead to negative outcomes, or increase positive behaviors like exercise (which then has additional physical health benefits) or engaging in hobbies (i.e., activities that are enjoyable encourage positive emotions).

Finally, internal resources such as self-care, hope [51] and acceptance, and spirituality also helped participants to cope. All are commonly used coping strategies and are generally considered examples of positive coping [59, 60]. Acceptance in particular may be related to wellbeing, and its common usage in the current study suggests it may be a good target for future interventions.

### Strengths and limitations

This study was exploratory and included a small number of participants, and therefore may not be generalizable. Though the number of participants aligns with previous research on sample size needed for data saturation, and though the these presented in this study were common within multiple study participants, it is possible that the themes addressed in this study are non-exhaustive. Additionally, interviews were only conducted with TB survivors who were still engaged with the larger TB Sequel [39] study and therefore excluded participants who may be lost to follow-up because their financial vulnerability prevented continued visits. Our findings do, however, align with related research and provide insight into the experiences of TB survivors, and therefore may provide insight into future directions for providing support and relief to TB survivors, such as virtual support groups when social isolation is necessary.

## Conclusion

The results of this study provide evidence that many of the stressors experienced by TB survivors during their diagnosis and treatment recurred during the COVID-19 pandemic, and so just as participants were recovering from TB they were forced once again to manage socioeconomic and physical stressors like financial insecurity and fears for physical health. Similarly, participants tended to tap into the same resources they used while they were ill with TB when confronting the COVID-19 pandemic. Social support in particular protected against one of the strongest indicators of TB disease burden, socioeconomic status, and future public health research should focus on fostering and maintaining a strong network of social support for TB survivors, including families and friends, other survivors, and health providers in continuing care. Additionally, research should address improved communication between patients and health professionals and economic pathways to pandemic containment that does not create additional or renewed burdens for the most vulnerable. Finally, the nuance associated with the contextual nature of help or harm in isolation should be explored.

## Data Availability

The datasets used and/or analyzed during the current study available from the corresponding author on reasonable request.

## References

[1] Centers for Disease Control and Prevention (CDC). Tuberculosis (TB) - Basic TB Facts. Atlanta: U.S. Department of Health and Human Services. 2016. https://www.cdc.gov/tb/topic/basics/default.htm. Accessed 13 Feb 2023.

[2] Kanabus A. TB in South Africa - Burden, strategic plan, key populations. Information about tuberculosis, GHE. 2022. https://tbfacts.org/tb-south-africa/. Accessed 13 Feb 2023.

[3] Isislow H. Tuberculosis remains disease of concern in South Africa. Anadolu Agency. 1 Oct 2022. https://www.aa.com.tr/en/africa/tuberculosis-remains-disease-of-concern-in-south-africa/2469695. Accessed 13 Feb 2023.

[4] Global Tuberculosis Programme. Global tuberculosis report 2022. Geneva: World Health Organization (WHO). 2023. https://www.who.int/publications/i/item/9789240061729. Accessed 13 Feb 2023.

[5] Karim SSA, Churchyard GJ, Karim QA, Lawn SD (2009). HIV infection and tuberculosis in South Africa: an urgent need to escalate the public health response. The Lancet.

[6] Addo J, Pearce D, Metcalf M, Lundquist C, Thomas G, Barros-Aguirre D (2022). Living with tuberculosis: a qualitative study of patients’ experiences with disease and treatment. BMC Public Health.

[7] Cramm JM, Koolman X, Møller V, Nieboer AP (2011). Socio-economic status and self-reported tuberculosis: a multilevel analysis in a low-income township in the Eastern Cape. South Africa. J Public Health Afr.

[8] Harling G, Ehrlich R, Myer L (2008). The social epidemiology of tuberculosis in South Africa: a multilevel analysis. Soc Sci Med 1982.

[9] Kallon II, Colvin CJ (2022). A qualitative exploration of continuity of TB care in clinics after discharge from hospitals in Cape Town, South Africa. BMC Health Serv Res.

[10] Francis D, Webster E (2019). Poverty and inequality in South Africa: critical reflections. Dev South Afr.

[11] Thungana Y, Wilkinson R, Zingela Z (2022). Comorbidity of mental ill-health in tuberculosis patients under treatment in a rural province of South Africa: a cross-sectional survey. BMJ Open.

[12] Dara M, Kuchukhidze G, Yedilbayev A, Perehinets I, Schmidt T, Van Grinsven WL (2021). Early COVID-19 pandemic’s toll on tuberculosis services, WHO European Region, January to June 2020. Euro Surveill Bull Eur Sur Mal Transm Eur Commun Dis Bull.

[13] Chudasama YV, Gillies CL, Zaccardi F, Coles B, Davies MJ, Seidu S (2020). Impact of COVID-19 on routine care for chronic diseases: a global survey of views from healthcare professionals. Diabetes Metab Syndr.

[14] Fei H, Yinyin X, Hui C, Ni W, Xin D, Wei C (2020). The impact of the COVID-19 epidemic on tuberculosis control in China. Lancet Reg Health West Pac.

[15] Pan American Health Organization (PAHO). Tuberculosis deaths and disease increase during the COVID-19 pandemic. PAHO/WHO. 27 Oct 2022. https://www.paho.org/en/news/27-10-2022-tuberculosis-deaths-and-disease-increase-during-covid-19-pandemic. Accessed 13 Feb 2023.

[16] Millones AK, Lecca L, Acosta D, Campos H, Del Águila-Rojas E, Farroñay S (2022). The impact of the COVID-19 pandemic on patients’ experiences obtaining a tuberculosis diagnosis in Peru: a mixed-methods study. BMC Infect Dis.

[17] World Health Organization (WHO). COVID-19 significantly impacts health services for noncommunicable diseases: Geneva: World Health Organization. 1 Jun 2020. https://www.who.int/news/item/01-06-2020-covid-19-significantly-impacts-health-services-for-noncommunicable-diseases. Accessed 13 Feb 2023.

[18] Shariq M, Sheikh JA, Quadir N, Sharma N, Hasnain SE, Ehtesham NZ (2022). COVID-19 and tuberculosis: the double whammy of respiratory pathogens. Eur Respir Rev off J Eur Respir Soc.

[19] Wang Q, Guo S, Wei X, Dong Q, Xu N, Li H (2022). Global prevalence, treatment and outcome of tuberculosis and COVID-19 coinfection: a systematic review and meta-analysis (from November 2019 to March 2021). BMJ Open.

[20] Mousquer GT, Peres A, Fiegenbaum M (2021). Pathology of TB/COVID-19 co-infection: the phantom menace. Tuberc Edinb Scotl.

[21] Eike D, Hogrebe M, Kifle D, Tregilgas M, Uppal A, Calmy A (2022). How the COVID-19 pandemic alters the Landscapes of the HIV and Tuberculosis Epidemics in South Africa: a case study and future directions. Epidemiol Basel Switz.

[22] World Health Organization (WHO). Global - South Africa. World Health Organization. 2023. https://covid19.who.int/region/afro/country/za. Accessed 13 Feb 2023.

[23] The Stop TB Partnership. The impact of COVID-19 on the TB epidemic - A community perspective. Geneva: The Stop TB Partnership. https://www.stoptb.org/impact-of-covid-19-tb-epidemic-community-perspective. Accessed 13 Feb 2023.

[24] Beyene NW, Sitotaw AL, Tegegn B, Bobosha K (2021). The impact of COVID-19 on the tuberculosis control activities in Addis Ababa. Pan Afr Med J.

[25] Abdool Karim Q, Baxter C (2022). COVID-19: impact on the HIV and Tuberculosis Response, Service Delivery, and Research in South Africa. Curr HIV/AIDS Rep.

[26] Benade M, Long L, Meyer-Rath G, Miot J, Evans D, Tucker J-M (2022). Reduction in initiations of drug-sensitive tuberculosis treatment in South Africa during the COVID-19 pandemic: analysis of retrospective, facility-level data. PLOS Glob Public Health.

[27] Meo SA, Abukhalaf AA, Alomar AA, AlMutairi FJ, Usmani AM, Klonoff DC (2020). Impact of lockdown on COVID-19 prevalence and mortality during 2020 pandemic: observational analysis of 27 countries. Eur J Med Res.

[28] Haider N, Osman AY, Gadzekpo A, Akipede GO, Asogun D, Ansumana R (2020). Lockdown measures in response to COVID-19 in nine sub-saharan african countries. BMJ Glob Health.

[29] Violato C, Violato EM, Violato EM (2021). Impact of the stringency of lockdown measures on covid-19: a theoretical model of a pandemic. PLoS ONE.

[30] McIntosh A, Bachmann M, Siedner MJ, Gareta D, Seeley J, Herbst K (2021). Effect of COVID-19 lockdown on hospital admissions and mortality in rural KwaZulu-Natal, South Africa: interrupted time series analysis. BMJ Open.

[31] Vanleeuw L, Zembe-Mkabile W, Atkins S (2022). Falling through the cracks: increased vulnerability and limited social assistance for TB patients and their households during COVID-19 in Cape Town, South Africa. PLOS Glob Public Health.

[32] Cilloni L, Fu H, Vesga JF, Dowdy D, Pretorius C, Ahmedov S (2020). The potential impact of the COVID-19 pandemic on the tuberculosis epidemic a modelling analysis. EClinicalMedicine.

[33] Kant S, Tyagi R (2021). The impact of COVID-19 on tuberculosis: challenges and opportunities. Ther Adv Infect Dis.

[34] Ask Afrika. *COVID-19 tracker: A gender report on South Africa* [PowerPoint Presentaton]. South Africa adjusted level 3 lockdown week 1, 2021 results. n.d. https://www.askafrika.co.za/wp-content/uploads/2021/05/Ask-Afrika-COVID-19-Omnibus-Week-1-2021-GENDER.pdf. Accessed 9 Feb 2023.

[35] The National Institute for Communicable Diseases of South Africa. Impact of COVID-19 interventions on TB testing in South Africa. 10. May 2020. https://www.nicd.ac.za/wp-content/uploads/2020/05/Impact-of-Covid-19-interventions-on-TB-testing-in-South-Africa-10-May-2020.pdf. Accessed Feb 9, 2023.

[36] Human Sciences Research Council (HSRC). *HSRC responds to the COVID-19* outbreak [PowerPoint Presentation]. Department of Science and Innovation, South Africa. n.d. https://hsrc.ac.za/uploads/pageContent/11529/COVID-19%20MASTER%20SLIDES%2026%20APRIL%202020%20FOR%20MEDIA%20BRIEFING%20FINAL.pdf. Accessed 9 Feb 2023.

[37] Loveday M, Hlangu S, Larkan L-M, Cox H, Daniels J, Mohr-Holland E (2021). This is not my body: therapeutic experiences and post-treatment health of people with rifampicin-resistant tuberculosis. PLoS ONE.

[38] TB Sequel Network. TB Sequel Project. : Research, Capacity Development, Networking. TB Sequel Network. n.d. https://www.https://www.tbsequel.org/tb-sequel-network/. Accessed 2 Dec 2022.

[39] Rachow A, Ivanova O, Wallis R, Charalambous S, Jani I, Bhatt N (2019). TB sequel: incidence, pathogenesis and risk factors of long-term medical and social sequelae of pulmonary TB – a study protocol. BMC Pulm Med.

[40] Carlitz RD, Makhura MN (2021). Life under lockdown: illustrating tradeoffs in South Africa’s response to COVID-19. World Dev.

[41] Mosiane M, Shabalala N, Ruch W, Khumalo R. Results from Wave 2 survey on the impact of the COVID-19 pandemic on employment and income in South Africa. Statistics South Africa; 2021.

[42] Naudé W, Cameron M (2021). Failing to pull together: South Africa’s troubled response to COVID-19. Transform Gov People Process Policy.

[43] Kessler RC, Andrews G, Colpe (2002). Short screening scales to monitor population prevalences and trends in non-specific psychological distress. Psychol Med.

[44] Andrews G, Slade T (2001). Interpreting scores on the Kessler Psychological Distress Scale (k10). Aust N Z J Public Health.

[45] Creswell JW (2012). Educational research: planning, conducting, and evaluating quantitative and qualitative research.

[46] Englund C, Olofsson AD, Price L (2018). The influence of sociocultural and structural contexts in academic change and development in higher education. High Educ.

[47] Langer PC (2016). The Research Vignette: reflexive writing as interpretative representation of qualitative Inquiry—A Methodological Proposition. Qual Inq.

[48] Jasinski L, Nokkala T, Juusola H (2021). Reflecting on the value of vignettes in higher education research: toward a preliminary typology to guide future usage. Eur J High Educ.

[49] Spalding NJ, Phillips T (2007). Exploring the use of vignettes: from validity to trustworthiness. Qual Health Res.

[50] Hennink M, Kaiser BN (2022). Sample sizes for saturation in qualitative research: a systematic review of empirical tests. Soc Sci Med.

[51] Daftary A, Mondal S, Zelnick J, Friedland G, Seepamore B, Boodhram R (2021). Dynamic needs and challenges of people with drug-resistant tuberculosis and HIV in South Africa: a qualitative study. Lancet Glob Health.

[52] Fritz J, Stochl J, Fried EI, Goodyer IM, van Borkulo CD, Wilkinson PO (2019). Unravelling the complex nature of resilience factors and their changes between early and later adolescence. BMC Med.

[53] Mbunge E (2020). Effects of COVID-19 in south african health system and society: an explanatory study. Diabetes Metab Syndr Clin Res Rev.

[54] Kim AW, Burgess R, Chiwandire N, Kwinda Z, Tsai AC, Norris SA (2021). Perceptions, risk and understandings of the COVID-19 pandemic in urban South Africa. South Afr J Psychiatry SAJP J Soc Psychiatr South Afr.

[55] Touré AA, Magassouba AS, Camara G, Doumbouya A, Cissé D, Barry I (2022). Health-Related Quality of Life of Tuberculosis patients during the COVID-19 pandemic in Conakry, Guinea: a mixed methods study. Trop Med Infect Dis.

[56] Sunjaya DK, Paskaria C, Pramayanti M, Herawati DMD, Parwati I (2022). The magnitude of anxiety and depressive symptoms among tuberculosis patients in Community Health Centers setting during the peak of COVID-19 pandemic. J Multidiscip Healthc.

[57] Bolak Boratav H, Karslıoğlu İnal S, Küey L. The experience of receiving a tuberculosis diagnosis: A qualitative exploration. Nesne. 2021;9(19):1-18. 10.7816/nesne-09-19-01.

[58] Laxmeshwar C, Steward AG, Dalal A, Kumar AMV, Kalaiselvi S (2019). Beyond cure and ‘treatment success’: quality of life of patients with multi-drug resistant tuberculosis. Int J Tuberc Lung Dis.

[59] Girma A, Ayalew E, Mesafint G (2021). Covid-19 pandemic-related stress and coping strategies among adults with chronic disease in Southwest Ethiopia. Neuropsychiatr Dis Treat.

[60] Umucu E, Lee B (2020). Examining the impact of COVID-19 on stress and coping strategies in individuals with disabilities and chronic conditions. Rehabil Psychol.

